# The Neural Basis of Event Simulation: An fMRI Study

**DOI:** 10.1371/journal.pone.0096534

**Published:** 2014-05-02

**Authors:** Yukihito Yomogida, Motoaki Sugiura, Yoritaka Akimoto, Carlos Makoto Miyauchi, Ryuta Kawashima

**Affiliations:** 1 Tamagawa University Brain Science Institute, Tokyo, Japan; 2 Japan Society for the Promotion of Science, Tokyo, Japan; 3 Department of Functional Brain Imaging, Institute of Development, Aging and Cancer, Tohoku University, Sendai, Japan; 4 International Research Institute of Disaster Science, Tohoku University, Sendai, Japan; 5 Smart Ageing International Research Centre, Institute of Development, Aging and Cancer, Tohoku University, Sendai, Japan; 6 Division of Developmental Cognitive Neuroscience, Institute of Development, Aging and Cancer, Tohoku University, Sendai, Japan; University of Udine, Italy

## Abstract

Event simulation (ES) is the situational inference process in which perceived event features such as objects, agents, and actions are associated in the brain to represent the whole situation. ES provides a common basis for various cognitive processes, such as perceptual prediction, situational understanding/prediction, and social cognition (such as mentalizing/trait inference). Here, functional magnetic resonance imaging was used to elucidate the neural substrates underlying important subdivisions within ES. First, the study investigated whether ES depends on different neural substrates when it is conducted explicitly and implicitly. Second, the existence of neural substrates specific to the future-prediction component of ES was assessed. Subjects were shown contextually related object pictures implying a situation and performed several picture–word-matching tasks. By varying task goals, subjects were made to infer the implied situation implicitly/explicitly or predict the future consequence of that situation. The results indicate that, whereas implicit ES activated the lateral prefrontal cortex and medial/lateral parietal cortex, explicit ES activated the medial prefrontal cortex, posterior cingulate cortex, and medial/lateral temporal cortex. Additionally, the left temporoparietal junction plays an important role in the future-prediction component of ES. These findings enrich our understanding of the neural substrates of the implicit/explicit/predictive aspects of ES-related cognitive processes.

## Introduction

To cope with dynamically changing physical and social environments, both current situations and future transitions must be inferred from fractionally perceived environmental information. Recently, such situational inferences have been proposed to be accomplished when partially perceived event features are associated in the brain to represent the whole situation [1]–[3]. Those event features include information concerning objects, agents, actions, mental states, and places that constitute a situation. By matching those event features with the event knowledge constructed through the accumulation of experience, the most likely situation is inferred. At the same time, other event features likely to appear in that situation but not perceived at that moment are also inferred based on the event knowledge. Via such completion inference, the whole situation is represented in the brain as a simulation. The situational inference process explained above is called event simulation (ES). ES is thought to provide a common basis for various cognitive processes. Those include simple perceptual prediction, situational understanding/prediction previously termed “event schema/script”, or complex social cognition such as “theory of mind” or trait inference [2], [4]. Thus, clarifying the detailed neural basis of ES would contribute to our understanding of these cognitive processes.

Although previous studies have assessed the neural basis underlying ES-related cognitive processes, such as language-based situational understanding [5]–[9] and social cognition (e.g., theory of mind (TOM) and trait inference) [10]–[20], no study has clarified the ES core, the process in which event features are integrated into a coherent representation of a situation. A few recent studies assessed this ES core more directly [21]–[26]. In these studies, event features (e.g., objects, agents, and backgrounds) that constitute a situation were presented in words or pictures, and the brain regions that respond to successful integration of these event features were examined. These studies demonstrated that the ES core process recruits the medial prefrontal cortex (MPFC), retrosplenial cortex (RSC), inferior parietal cortex (IPL), dorso-lateral prefrontal cortex (DLPFC), ventro-lateral prefrontal cortex (VLPFC), parahippocampal cortex (PHC), middle temporal gyrus (MTG), and temporal pole (TP).

Although previous studies have assessed the neural basis of ES as a whole, critical ES subdivisions and the neural basis of those subdivisions have not yet been clarified. For example, whether ES recruits similar or different neural substrates when occurring explicitly versus implicitly remains unclear. ES is sometimes conducted without an explicit intention to do so; this implicit aspect of ES has great significance. For example, in daily life, an individual will usually spontaneously notice his or her own situation. In this case, ES occurs implicitly. On the other hand, although this is often the case in a laboratory experiment, it is rare that an individual will need to explicitly understand a situation in daily life. When “implicit”, the aforementioned process is not assumed to be unconscious. Instead, it is characterized by its spontaneous activation via bottom-up input from the environment, regardless of explicit intentions to do so. Another example of the importance of the implicit aspect of ES is the fact that social phenomena such as stereotypes or prejudices are problematic due to their implicit nature [27]–[29]. Because these phenomena depend on ES-based trait inference, clarifying the neural mechanisms underlying the implicit aspects of ES would contribute to understanding these social phenomena. In fact, implicit aspects of various cognitive processes and their neural substrates have attracted immense attention in recent years [27], [29]–[33]. However, previous studies have not differentiated the neural basis of implicit ES from that of explicit ES. Of the above-mentioned studies that directly assessed the ES core, that by Bar et al. employed only an implicit task [21]. Other studies employed only explicit tasks [22]–[26]. So, there is an urgent need to differentiate the neural basis of implicit and explicit ES by conducting a comparison in a unified experimental framework. In the present study, we differentiated between the neural substrates of implicit and explicit ES by assuming that two cognitive processes underpin implicit and explicit ES. The first process is recruited by implicit ES (implicit ES process) and is also recruited when ES is conducted explicitly, thus providing a common ground for implicit and explicit ES. The second process is recruited together with the implicit ES process in the explicit ES (explicit ES process). We differentiated brain regions specific to those processes using the following paradigm. In one condition, subjects were shown a picture set of contextually irrelevant objects and performed an object-name-matching task. Unknown to the subjects, contextually related objects, indicating a situation, were occasionally inserted to induce implicit ES. Brain regions that increased their activity in response to this insertion were assumed to be the neural basis of the implicit ES process. In the second condition, subjects were explicitly instructed to infer the situation indicated by objects to demonstrate explicit ES. Brain activation induced in the explicit condition was compared with that induced in the implicit condition to clarify the neural basis of the explicit ES process. Based on previous findings noted above, it was expected that ES as a whole would recruit the MPFC, RSC, IPL, DLPFC/VLPFC, PHC, MTG, and TP. More importantly, it was expected that implicit and explicit ES processes would recruit different subsets of these brain regions.

Another important aspect of ES is the future-prediction component, but the neural basis underlying this component is not yet known. To cope with dynamically changing environments, not only must the current situation be inferred but the possible future situation must also be predicted. This predictive component plays an essential role in ES. Previous studies that assessed the neural basis of future prediction emphasized that common brain networks are recruited as in episodic memory recall, including the hippocampus, PHC, MPFC, VLPFC, RSC, precuneus, posterior cingulate cortex (PCC), and the temporo-parietal junction (TPJ) [34]–[42]. Based on these findings, it has been suggested that future prediction and episodic memory recall both depend on a common ‘scene construction’ process [43], [44]. This process is similar to the completion inference process in ES, as it integrates event features into a coherent situational representation. In addition to this completion inference process, however, future prediction additionally demands representing unexperienced future situations as hypothetical ones. Previous studies have not dissociated this key component of future prediction from completion inference itself. Consequently, the specific neural substrate of this component is still unknown. As for the candidate neural substrates of this component, we expected that the TPJ plays a primary role among the regions previously indicated to be involved in future prediction. This is because the TPJ has been consistently indicated to be involved in representing hypothetical perspective or perspective differences in previous studies on TOM, perspective taking, and self-transcendence [45]–[51]. In the present study, to extract brain regions specific to representing a hypothetical situation in future prediction, the following paradigm was used. In addition to the above-explained explicit task condition in which subjects were asked to infer a situation indicated by objects, we also used a future-prediction condition in which subjects were asked to predict future situations likely to happen after those indicated by the objects had already occurred. By comparing the brain activity induced under these two task conditions, this study aimed to clarify the neural basis of the future-prediction component of ES.

## Materials and Methods

### Subjects

Twenty-three healthy volunteers with no psychiatric or neurological history participated in the present study. All were right-handed as assessed using Edinburgh Handedness Inventory [52]. Written informed consent was obtained from all subjects prior to their participation. Data from one subject were excluded due to head motion and from two subjects because they fell asleep in the scanner. Thus, data of 20 subjects (nine female and 11 male) with an average age of 20.65 years (SD = 2.13) were analyzed. The current study was approved by the ethics committee of the Tohoku University Graduate School of Medicine.

### Cognitive task detail

**Figure 1:**
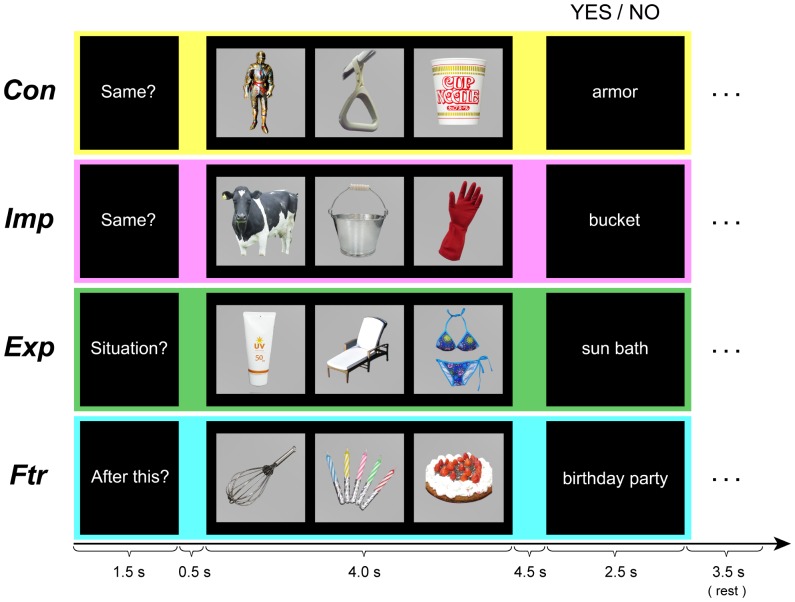
Schematic depiction of a trial for each task and condition. In the Object task (cue “Same?”), the subject answered whether one of the three objects presented was congruent with the subsequently presented target word (i.e., object name). Three objects presented in the Con condition were contextually unrelated, and those in the Imp condition were contextually related and indicated a situation. In the Situation task (cue “Situation?”), the subject answered whether the target word was properly depicting the situation indicated by the object pictures. In the Future-prediction task (cue “After this?”), the subject answered whether the target word was properly depicting possible future events of the indicated situation.

Subjects participated in the following three types of matching tasks in the scanner (Fig. 1). The first task was the Object task (starts with the cue “Same?”). Following the cue, a picture set comprising three object pictures was presented. After a 4.5-sec delay period, an object name (target) was presented. Subjects were instructed to judge the congruency of the picture set and target object name. If the object name matched one of the three objects the subjects observed in the picture set, they had to respond “yes” by pushing a button with their right index finger. Otherwise, they were told to answer “no” by pushing another button with their right middle finger. Unknown to subjects, this task included two conditions. In half of the trials, the three objects in the picture set were contextually unrelated (control, Con), whereas in the remaining half of the trials, these object pictures were contextually related and indicated a situation (e.g., milk a cow; Fig. 1) to trigger an implicit ES (implicit, Imp). The second task was the Situation task (starts with the cue “Situation?”) in which subjects observed an object picture set similar to the Imp of the Object task and a target word depicting a situation. Subjects were asked to explicitly understand the situation indicated by the pictures and to answer whether this situation matched the target word (explicit, Exp). The third task was the Future-prediction task (starts with the cue “After this?”). Again, an object picture set similar to those used in the Imp and Exp conditions was presented to subjects. This time, the subject was instructed to understand the current situation indicated by the pictures (e.g., making a birthday cake) and then infer the possible future situation (e.g., starting birthday party) likely to occur afterword (future prediction, Ftr). The subject responded “yes” if the target word correctly depicted a possible future situation. In all the above conditions, the time course of trials was identical. A 1.5-s cue was followed by a 0.5-s fixation delay and then the object picture set was presented for 4-s followed by a 4.5-s delay period. Subsequently, a target word was presented for 2.5-s followed by a 3.5-s inter-trial rest period.

To extract the activation associated with the ES process itself (i.e., the activation induced during the picture presentation and subsequent delay) and the activation associated with the target response (i.e., the activation induced after target presentation) separately, “incomplete” trials, which were interrupted after the 4.5-s delay period by showing an “X” sign instead of a target word, were introduced. Subjects were instructed that if they observed the “X” sign, they did not need to respond to any question and were instructed to wait for the next trial. Half of the trials in each condition (Con, Imp, Exp, and Ftr) were randomly presented as incomplete so that subjects could not predict the interruption. The purpose of including these incomplete trials was to increase the orthogonality of the hemodynamic response models between the ES and target response phases. Without incomplete trials, those two models would have considerable degrees of correlation because the ES and response phases occur in a fixed order with a small time separation relative to the time constant of the hemodynamic response function. By including incomplete trials, we were able to observe what happened when the target response phase did not follow the ES phase in half the trials, which allowed us to separate the neural response patterns of these two phases in the analysis. Because only the ES phase was included in the contrast for purposes of statistical testing, the current results are free from the effects related to the target response phase (e.g., recognition of target, matching decisions, semantic priming) that might occur in target processing.

Each subject underwent two task sessions with 48 trials each (12 trials for the conditions Con, Imp, Exp, and Ftr). Thus, 96 trials (Con: 24 trials; Imp: 24 trials; Exp: 24 trials; Ftr: 24 trials) were presented to each subject. Each condition (Con, Imp, Exp, and Ftr) appeared in a pseudo-randomized order. At the beginning of the experiment, all subjects underwent a practice session outside of the scanner and were assured that they could solve all tasks easily. In this practice session, 12 trials were presented for each of the Con, Exp, and Ftr conditions. In each condition, picture sets different from those used in the functional magnetic resonance imaging (fMRI) session were presented.

### Post-scanning questions

Following the scanning session, we assessed whether subjects noticed that object pictures were associated with a situation in the Imp condition to ensure that stimuli presented in the Imp condition elicited implicit ES even in the absence of an explicit task requirement. First, subjects were asked the following question: “Did you notice something when you were doing Object task?” Then, they were asked more directly, “Did you notice that sometimes object pictures were associated with a situation in the Object task?” They answered “yes” or “no”.

Additionally, we determined the extent to which subjects were familiar with the situations they saw in the Imp, Exp, and Ftr conditions. Subjects were asked to rate familiarity using a four-point scale [1 (“Not at all familiar”) to 4 (“Very familiar”)] to ensure that the results were not confounded by differences in familiarity across conditions.

### Stimulus preparation

We prepared the stimulus sets for the fMRI task paradigm described above in accordance with the following principles. First, stimulus sets were prepared so that all experimental conditions (Con, Imp, Exp, Ftr) involved the same object picture stimuli when averaged across subjects. Thus, observed differences in neural activity across conditions could not be attributed to differences in stimuli-related factors such as low-level visual processing, the object recognition process, or contextual information inherent in the objects themselves. Second, stimulus sets were prepared so that each experimental condition involved different object picture sets at the individual level. This was essential to avoid any noise caused by repetition of the stimulus in different conditions. To meet these criteria, 96 object picture sets in which objects were contextually related to a situation were prepared. The 96 picture sets were divided into four subsets (A, B, C, and D). Thus, each subgroup comprised 24 picture sets. Thereafter, scrambled versions of each subset (Ascr, Bscr, Cscr, and Dscr) were created by shuffling objects within each subset and making them contextually unrelated. Such contextually unrelated scrambled subsets were allocated to the Con condition so as to prevent recognition of the situation in this condition. The other three original subsets were allocated to the Imp, Exp, and Ftr conditions, respectively (e.g., Ascr-Con, B-Imp, C-Exp, D-Ftr). Allocation of those subsets was counterbalanced across subjects.

Specifically, the following two-stage preliminary experiments were conducted to create the stimulus set described above. Subjects were recruited from the same subject pool (i.e., students from the same university) used for the fMRI experiment. In all, 14 (5 female and 9 male) and 12 subjects (6 female and 6 male) who did not participate in the fMRI experiment participated in the first and second experiments, respectively. Information about the age of one subject in each experiment was missing due to registration failure, and data on the age of the remaining subjects in each experiment are provided below. The average ages for the first and second experiment were 20.92 years (SD = 1.44) and 19.36 years (SD = 0.92), respectively. In the first experiment, object picture sets from which both the current and future situations could easily be imagined were selected, yielding a total of 200 object picture sets. Object pictures were taken by digital camera or obtained from publicly available Internet sources. These objects were limited to non-human objects, such as commodities, vehicles, and animals. No human images (e.g., faces) were included except for ones that appeared as part of another object (e.g., magazine cover). Subjects viewed these 200 picture sets and answered the following two questions for each: 1) Q-scn: “Can you imagine a situation from this picture set?” and 2) Q-Ftr: “Can you imagine the future situation likely to occur after the situation indicated by this picture set?” Subjects answered these questions using a four-point scale [1 (“No, I can't imagine one at all”) to 4 (“Yes, I can imagine one easily”)]. To select picture sets that ranked highly on both questions, mean ratings for each question type were obtained for each picture set, picture sets were ranked according to the lower of the two rankings, and the 96 highest-scoring picture sets were chosen. The average subjects' Q-scn and Q-Ftr ratings for these selected picture sets were 3.94±0.08 [mean ± standard deviation (SD)] and 3.83±0.10, respectively. Subsequently, the 96 picture sets were divided into four subsets (A, B, C, and D) while balancing Q-scn and Q-Ftr scores between them.

Next, scrambled versions of the object picture sets created above (Ascr, Bscr, Cscr, and Dscr) were generated, and we determined whether these scrambled versions would prevent recognition of the situation. In the second experiment, subjects viewed both the original and scrambled picture sets and rated the likelihood of imagining a situation from those picture sets using the same four-point scale described above. The average ratings of the original and scrambled picture sets were 3.85±0.09 and 1.16±0.15, respectively. A paired *t*-test indicated that scrambled picture sets scored significantly lower than the originals (*p* = 5.22×10^−16^).

Among the final 96 picture sets, 41 (42.7%) depicted a social situation. Here, a social situation is defined as a situation in which human interaction or communication is evident [53] (e.g., wedding dress + tuxedo + wedding cake  =  wedding ceremony). As we noted above, stimulus sets were prepared so that all experimental conditions (Imp, Exp, and Ftr) involved the same stimuli sets when averaged across subjects. Thus, the proportion of social and non-social situations was totally balanced across these conditions.

### fMRI measurement and image preprocessing

Thirty-three gradient-echo images (echo time  = 25 ms, flip angle  = 78°, slice thickness  = 3 mm, slice gap  = 1 mm, field of view  = 200 mm, matrix size  = 64×64) covering the whole brain were acquired at a repetition time of 2000 ms using an echo planar sequence and a 3-T magnetic resonance scanner (Achieva Quasar Dual, Philips Medical Systems; Best, The Netherlands).

For each subject, data were acquired in two scanning sessions. Excluding the first two “dummy” volumes for stabilization of the T1-saturation effect, 404 volumes were acquired in each fMRI session. The following preprocessing procedures were performed using Statistical Parametric Mapping (SPM8) software (Wellcome Department of Imaging Neuroscience; London, UK) implemented in MATLAB R2009b (MathWorks; Natick, MA, USA) for whole brain analysis: correction for head motion, adjustment of acquisition timing across slices, spatial normalization using the MNI template, and smoothing using a Gaussian kernel with a full width at a half-maximum of 5 mm.

### fMRI data analysis

The following series of subtraction analyses were conducted to differentiate the brain regions specific to the implicit and explicit ES processes and to clarify the neural bases specific to future prediction. First, brain regions specific to the implicit ES process were evaluated using the contrast (Imp–Con). In the Object task, subjects were engaged in the same task in both the Con and Imp conditions. However, in the Imp condition, object pictures were contextually indicating a situation, thereby inducing implicit ES. Hence, it was assumed that differential brain activation between these conditions reflected the implicit ES process. Second, brain regions specific to the explicit ES process were evaluated using the contrast (Exp–Imp). As the same object picture sets were shown to subjects in both the Exp and Imp conditions, these conditions commonly induced the implicit ES process. Conversely, only in the Exp condition was the explicit ES process additionally induced because subjects were required to conduct explicit ES in this condition. Hence, it was assumed that the differential brain activation between the Exp and Imp conditions reflects the explicit ES process. Finally, brain regions specific to future prediction were evaluated using the contrast (Ftr–Exp). The same object picture sets were shown to subjects in both the Ftr and Exp conditions. Similarly, both conditions required subjects to explicitly understand the situations indicated by those stimulus sets. Conversely, only in the Ftr condition were subjects additionally required to infer a future situation that is likely to occur after the current situation indicated by the stimulus. Thus, it was assumed that the differential brain activation between the Ftr and Exp conditions reflects a future-prediction component of ES.

To conduct the series of subtraction analyses depicted above, a conventional two-level approach was adopted using SPM8. A set of regressors was generated by convolving a canonical hemodynamic response function provided by SPM8 with a series of epochs. For each condition (Con, Imp, Exp, and Ftr), the period from the time of picture set presentation to the end of the subsequent 4.5-s delay was modeled as the regressor of interest. Additionally, the target (or “X” mark) and inter-trial rest period were modeled individually as regressors of no interest. These regressors of no interest were not included in contrasts for statistical inference. A voxel-by-voxel multiple regression analysis of these regressors was applied to the preprocessed images for each subject. Statistical inference on contrasts of parameter estimates was then performed at the second-level between-subjects (random effects) model using a one-sample *t*-test. The statistical threshold was set to *p*<0.001 for height and corrected to *p*<0.05 for multiple comparisons using cluster size assuming the whole brain as the search volume [54]. Additionally, results surviving a threshold of *p*<0.001 without multiple comparisons in two regions are reported here; namely, the PHC and the TPJ. This is because their contribution to ES was expected based on previous findings (see the Introduction).

To reject the possibility that the brain activation detected in the main subtraction analysis was due to the difference in task difficulty between conditions, an additional parametric modulation analysis using response times (RTs) as an explanatory variable was conducted. It was reasoned that if the brain activation detected in the main subtraction analysis reflected the difference in task difficulty or general cognitive load commonly recruited in the two conditions included in a contrast, then the activation of these regions should be correlated with RTs in the relative control condition of that contrast. The direction of the correlation should be positive if RTs in the target condition were longer than those in the relative control condition and vice versa. This possibility was evaluated as follows. A parametric modulation analysis was conducted that sought brain regions in which activity was correlated positively or negatively with RTs in each condition. This was performed by expanding the original model used in the main analysis by adding RTs for each condition as parametric modulators. Next, if RTs differed between the two conditions included in a contrast in the main analysis, we determined whether each peak voxel detected in that contrast also appeared as RT-correlated areas in the relative control condition at the statistical threshold of uncorrected *p*<0.05 in the second analysis.

**Figure 2:**
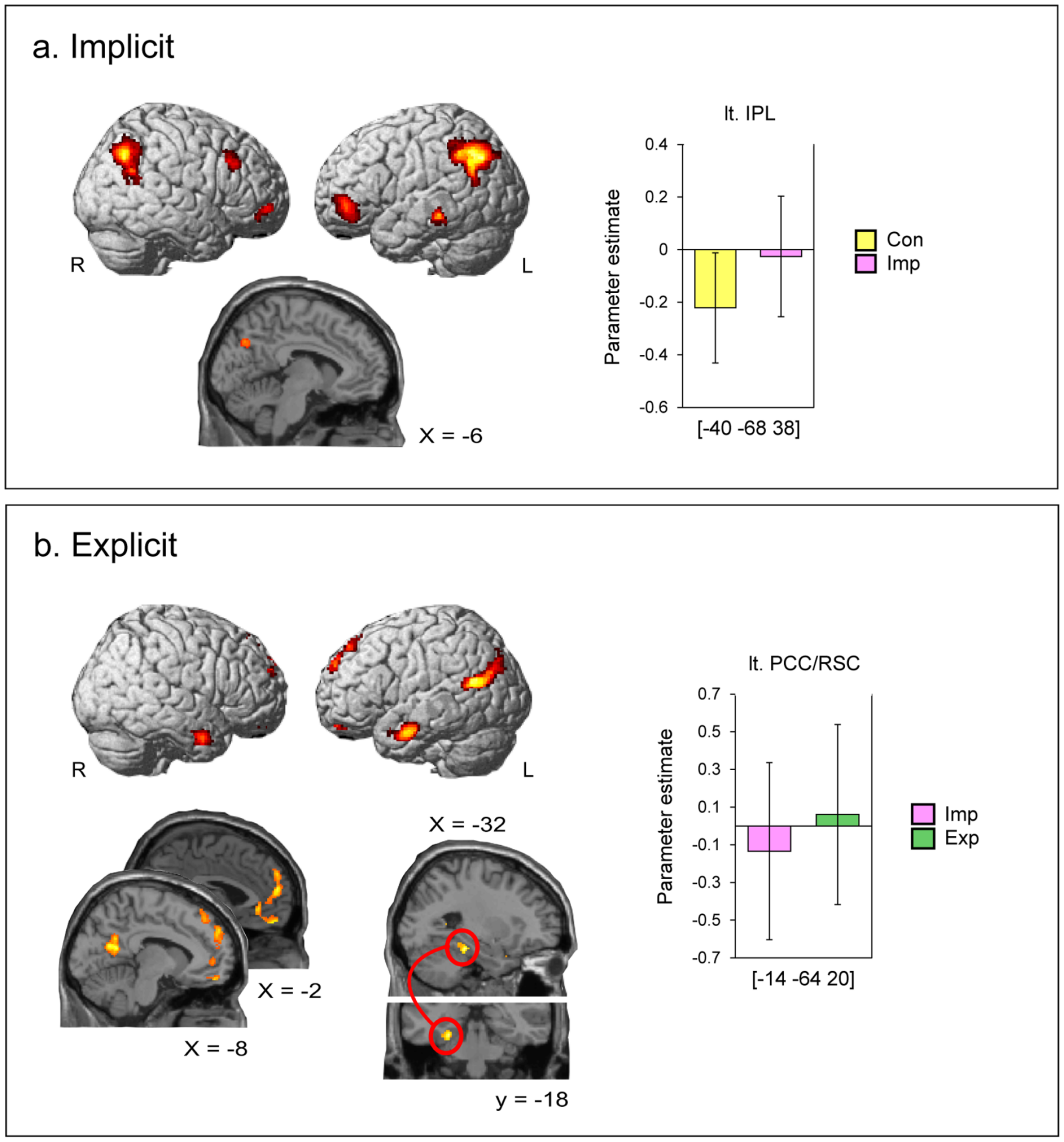
Activation areas specific to the implicit and explicit event simulation (ES) processes. All voxels except for the regions described below are significant at a statistical threshold of *p*<0.001, corrected to *p*<0.05 for multiple comparisons using the cluster size, assuming the whole brain as the search volume. The result of the left parahippocampal cortex in the explicit ES process is thresholded at *p*<0.001 (uncorrected). Error bars indicate standard deviations (SDs). IPL: inferior parietal lobule. PCC: posterior cingulate cortex. RSC: retrosplenial cortex. R: right. L: left. The coordinates in the Montreal Neurological Institute (MNI) standard space are indicated.

As the explicit ES process-specific brain regions revealed by the contrast (Exp – Imp) were well known social cognition-related regions (see results: Fig. 2*b*) [55], [56], we examined whether this result was affected by the social factor of task stimuli (i.e., whether the object picture set indicated a social or non-social situation). To clarify this issue, we conducted another additional analysis in which the effects of (Exp – Imp) in social events and that in non-social events were tested separately. First, trials in Imp and Exp conditions were split into ones that indicated social and non-social situations (i.e., Imp_social, Imp_nonsocial, Exp_social, and Exp_nonsocial), and modeled as separate regressors, respectively. Then, the two contrasts (Exp_social – Imp_social) and (Exp_nonsocial – Imp_nonsocial) were tested. If the original (Exp – Imp) comparison was not affected by social factors and rather reflected general situational inference load, then both new social and non-social contrasts would replicate the activation pattern seen in the original comparison. Considering that sample trial number in each condition was reduced in this analysis, the statistical threshold was set to p<0.005 without multiple comparisons.

The fMRI and behavioral data used in the above analysis are available to all interested researchers upon request (contact the corresponding author).

## Results

### Behavioral data

The accuracies of the conditions Con, Imp, Exp, and Ftr were 96.25±6.33%, 95.00±6.28%, 97.50±3.92%, and 89.58±9.32%, respectively. A one-way analysis of variance (ANOVA) indicated a significant effect of condition; *F*(3,76) = 5.8678 (*p*<0.01). *Post hoc* Bonferroni's multiple comparison tests revealed that this difference was due to the lower accuracy in the Ftr condition than in the Con (*p*<0.05) and Exp (*p*<0.05) conditions.

Second, the RTs for the Con, Imp, Exp, and Ftr conditions were 1059.62±214.51 ms, 998.61±197.03 ms, 1007.82±197.13 ms, and 1246.55±204.59 ms, respectively. A one-way ANOVA indicated a significant effect of condition; *F*(3,76)  = 39.418 (*p*<0.01). *Post hoc* Bonferroni's multiple comparison tests revealed that the RTs in the Ftr condition were significantly longer than were those in the Con (*p*<0.01), Imp (*p*<0.01), and Exp (*p*<0.01) conditions. Additionally, the RTs in the Con condition were longer than were those in the Imp condition (*p*<0.05). No statistically significant RT difference was found between the Imp and Exp or the Con and Exp conditions.

Post-scanning questions revealed that subjects noticed that object pictures were associated with a particular situation in Imp condition. In response to the first question, 16 of 20 subjects (80%) mentioned that they saw some object picture sets that indicated a situation in the Object task. This proportion increased to 100% when subjects were more directly asked, in the second question. This clearly indicates that implicit ES was elicited in the Imp condition even in the absence of an explicit task requirement.

Subjects' familiarity with situations presented in the Imp, Exp, and Ftr conditions were 2.73±0.72, 2.80±0.66, and 2.70±0.52, respectively. A one-way ANOVA revealed no statistically significant difference. Thus, the current results are not confounded by differences in familiarity.

### fMRI data

#### Implicit ES process-specific areas

Implicit ES process-specific regions were evaluated using the contrast (Imp–Con). A statistically significant activation was found in the bilateral IPL, left middle temporal gyrus (MTG), bilateral anterior VLPFC, right DLPFC, and bilateral precuneus (Fig. 2*a*; Table 1). In the right DLFPC region, the activation peak was located at the lower end of the middle frontal gyrus (MFG) whereas the cluster itself spread over both the MFG and inferior frontal gyrus. Because the 1^st^ cluster right IPL reflected task difficulty between conditions rather than implicit ES processes (see below), the activation profile of the 2^nd^ cluster/1^st^ peak (left IPL) is shown in Figure 2a.

**Table 1:** Clusters of activation.

contrast	brain region	x	y	z	*t* value	
*Implicit, [Imp - Control]*						
	rt. inferior parietal lobule	46	−64	38	8.78	
		40	−60	34	7.68	
		46	−54	46	5.02	§
	lt. inferior parietal lobule	−40	−68	38	8.48	
		−42	−48	38	7.27	
		−46	−60	44	7.24	
	lt. middle temporal gyrus	−56	−32	−10	6.79	
		−62	−36	−16	5.85	§
	lt. middle fronal gyrus	−40	46	2	6.64	
	lt. inferior frontal gyrus	−46	42	−2	5.97	
		−42	38	−12	5.08	
	rt. middle frontal gyrus	40	52	−6	5.42	
	rt. inferior frontal gyrus	42	46	−12	4.31	§
	rt. dorsolateral prefrontal cortex	50	28	34	5.18	
	lt. precuneus	−6	−72	36	4.99	
	rt. precuneus	4	−62	36	4.56	
*Explicit, [Exp - Imp]*						
	lt. posterior cingulate cortex	−14	−64	20	7.52	
	/retrosplenial cortex	−8	−56	26	5.23	
		−22	−54	8	4.33	
	lt. temporal pole	−54	2	−26	7.11	
		−48	10	−22	4.48	
	lt. TPJ	−40	−82	38	7.04	
		−56	−60	18	6.48	
		−50	−72	26	5.53	
	rt. dMPFC	8	56	24	6.06	
	lt. dMPFC	−2	56	16	6.03	
		−6	56	34	5.42	
	rt. temporal pole	54	2	−30	5.80	
		48	−8	−24	3.82	
	lt. vMPFC	−4	54	−18	5.78	
	lt. ACC	−2	36	−4	5.18	
		−4	42	−14	4.20	
	lt. parahippocampal gyrus*	−26	−32	−18	6.50	
*Future prediction, [Ftr - Exp]*						
	lt. TPJ**	−42	−52	22	5.31	
		−42	−48	12	4.19	

Clusters with significant activation associated with implicit, explicit, or future prediction.

Significance level: p<0.001 with cluster correction for multiple comparisons (p<0.05).

(* p<0.001 uncorrected, **p<0.001 with cluster correction for multiple comparisons [p<0.06, k = 133]).

§ : activation peaks met the exclusion criteria described in the Methods & Results sections.

Size: Numbers of voxels. t value: maximum t value at the peak voxels.

x,y,z: MNI coordinates of peak voxel; TPJ: temporo-parietal junction.

dMPFC: dorsal medial prefrontal cortex; vMPFC: ventral medial prefrontal cortex.

ACC: anterior cingulate cortex.

#### Explicit ES process-specific areas

Explicit ES process-specific regions were evaluated using the contrast (Exp–Imp). A statistically significant activation was found in the dorsal/ventral MPFC and adjacent ACC, bilateral TP, left PCC and adjacent RSC, and left TPJ (Fig. 2*b* and Table 1). Additionally, a significant activation was found in the left PHC at a threshold of *p*<0.001 (uncorrected). Although this region did not survive the multiple comparisons analysis, it is reported due to the hypothesis describing PHC involvement in ES, which was based on previous findings (see the Introduction). The activation profile of the 1^st^ cluster/1^st^ peak (left PCC/RS) is shown in Figure 2b.

#### Future-prediction-specific areas

**Figure 3:**
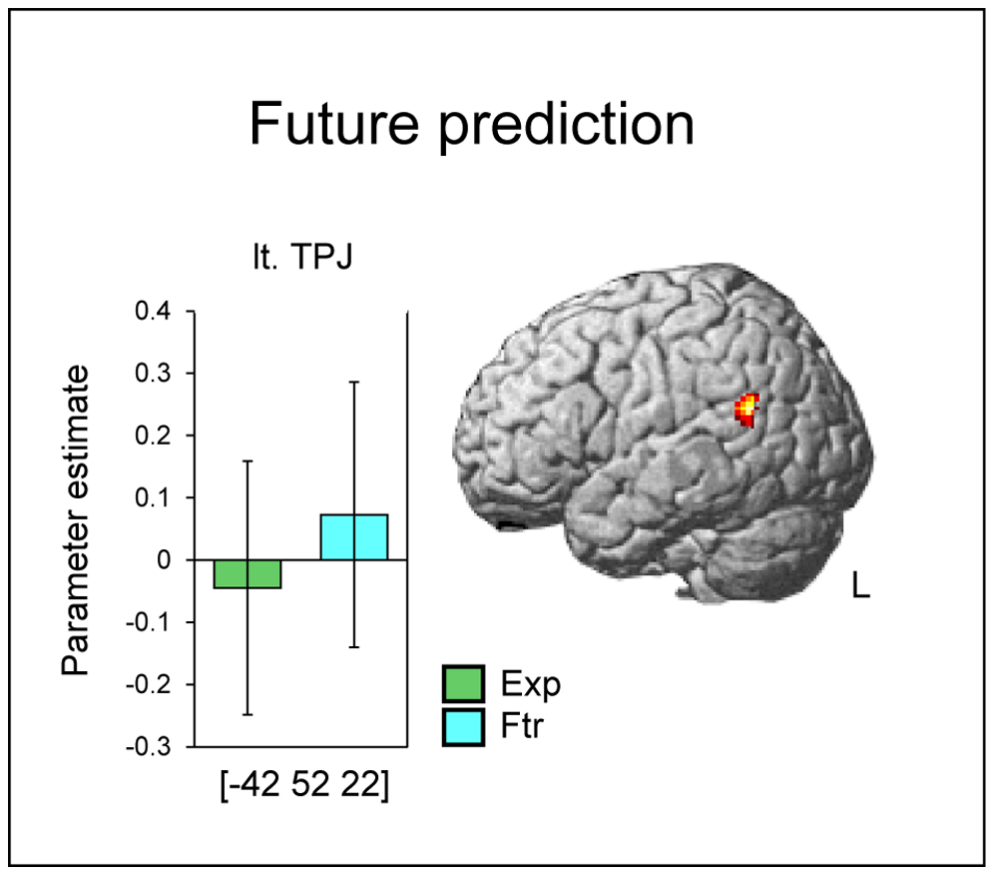
Activation areas specific to future prediction. The result is thresholded at *p*<0.001, corrected to *p*<0.06 (*k* = 133) for multiple comparisons. Error bars indicate SD. TPJ: temporoparietal junction. R: right. L: left. The coordinates in the MNI standard space are indicated.

Future-prediction-specific regions were evaluated using the contrast (Ftr–Exp). A statistically significant activation was found in the left TPJ at a threshold of an uncorrected *p*<0.001 (Fig. 3; Table 1). Although this region did not survive the multiple comparisons analysis, it is reported for two reasons: first, due to the hypothesis concerning TPJ involvement in future prediction (see the Introduction); second, the *p* value of this region after correction was close to significance (*p* = 0.06). The activation profile of the 1^st^ cluster/1^st^ peak (left TPJ) is shown in Figure 3.

#### Additional parametric modulation analysis

Behavioral data showed that RTs differed between conditions in the contrast (Ftr – Exp). RTs in the Ftr condition were longer than those in the Exp condition. If the brain activation revealed by the contrast (Ftr – Exp) was caused by increased ‘general’ task difficulty or cognitive load reflected in longer RTs, then the activity of these regions should have been greater when trial RTs were longer even within the Exp condition. In other words, the activity in these regions should have been positively correlated with trial RTs in the Exp condition. We looked into this possibility by conducting an additional parametric modulation analysis which sought the brain regions in which activity was positively correlated with trial RTs in the Exp condition (thresholded at uncorrected p<0.05) and assessed whether the left TPJ peak revealed by the (Ftr – Exp) contrast fell within these regions; this was not the case. Thus, the left TPJ activity reflected a qualitative difference in cognitive processes between the Ftr and Exp conditions (i.e., future prediction) rather than an increase in general task difficulty or cognitive load.

Similarly, RTs differed between conditions in the contrast (Imp - Con). This time, RT in the Imp condition was shorter than that in the Con condition. Thus, following similar logic as above, if brain activations revealed by the contrast (Imp – Con) just reflected a decrease in general task difficulty or cognitive load in the Imp condition, the activity in these regions should have been negatively correlated with RTs in the Con condition. We examined this possibility by conducting an additional parametric modulation analysis which sought the brain regions in which activity was negatively correlated with RTs in the Con condition (thresholded at uncorrected p<0.05) and assessed whether activation peaks found in the contrast (Imp – Con) fell within these regions. The right IPL, left MTG, and right anterior VLPFC met that criterion (Table 1; right-most column), indicating that these regions' activity could have reflected differences in general task difficulty or cognitive load between the Imp and Con conditions. In contrast, other regions revealed by the contrast (Imp – Con) reflected a qualitative difference in cognitive processes between these conditions (i.e., implicit ES process).

#### Additional analysis to assess the effect specific to social situations

Brain regions specific to the explicit ES process in social events and those specific to non-social events were separately evaluated by testing the contrasts (Exp_social – Imp_social) and (Exp_nonsocial – Imp_nonsocial), respectively. Both tests replicated similar activation patterns observed in the original (Exp – Imp) comparison (see Fig. S1). Thus, we suggest that the explicit ES process-specific regions we showed above (Fig. 2*b*) reflect general situational inference processes rather than any social situation-specific factors.

## Discussion

In the present study, the neural basis of ES, or the process by which partially perceived event features are associated in the brain to represent the whole situation, was clarified. Two major findings were revealed by this study. First, implicit and explicit ES processes depend on different neural substrates. Second, the future-prediction component of ES increases the activity of the left TPJ among those ES networks. These issues are discussed in detail in the following sections.

### Implicit versus explicit ES

The present results suggest that implicit and explicit ES processes depend on different neural substrates. The implicit ES process, which provides the common ground for implicit and explicit ES, recruits the left anterior VLPFC, right DLPFC, bilateral precuneus, and left IPL. Conversely, the explicit ES process, which characterizes the explicit ES, recruits the dorsal and ventral part of the MPFC and its adjacent ACC, left PHC, bilateral TP, left PCC/RSC regions, and left TPJ. In addition to the regions stated above, the right IPL, left MTG, and right anterior VLPFC were also associated with the implicit ES network. However, those regions reflected the difference in task difficulty between conditions in that contrast rather than the ES itself. Thus, those regions are not discussed.

The neural basis of implicit and explicit ES processes has not been distinguished thus far. The current findings provide the first evidence of such a dissociation and contribute to an understanding of the neural mechanisms underlying ES. Because ES provides the common basis for various cognitive processes from situational understanding to social cognition, clarifying its neural basis in terms of both implicit and explicit aspects is of great significance. For example, an understanding of the neural mechanisms underlying the implicit and explicit aspects of various social cognitive processes would be enriched. The difference between explicit and implicit processes in social cognition has been of great importance for many years, and clarification of the neural bases of these processes will have important ramifications [29], [57], [58]. The C-system/X-system model proposed by Lieberman *et al*. is well-known [32], [59]. This model comprises a list of brain regions recruited in implicit and explicit processes and was constructed by reviewing the literature on social cognition and related cognitive processes. In this model, the orbitofrontal cortex, basal ganglia, amygdala, lateral temporal cortex, and dorsal ACC regions are classified as the implicit/X-system. Conversely, the lateral prefrontal cortex, medial temporal lobe (MTL), rostral ACC/MPFC, and lateral/medial posterior parietal cortex (PPC) comprise the explicit/C-system. The current results provide additional information regarding this distinction of implicit/explicit systems from the viewpoint of ES. These findings confirm, in part, the present C-system/X-system model by showing that the explicit process recruits the MPFC, MTL, and PPC. Furthermore, within these regions, the lateral PPC was recruited not only by the explicit process but by the implicit process as well. The explicit and implicit processes activated different subregions within the lateral PPC. Similarly, it was found that different subregions in the medial PPC were recruited by the explicit and implicit processes, respectively. These findings will help to elaborate the current C-system/X-system model and might enrich our understanding of the neural substrates of social cognitive processes, particularly those such as stereotyping or prejudice, in which implicit aspects play a substantial role.

The regional distinction of the implicit/explicit network revealed here is supported by previous studies investigating ES-related cognitive processes. For example, the present study revealed that the left anterior VLPFC is engaged in the implicit process, and lesions in the VLPFC are known to reduce implicit stereotypical attitudes [60]. Because stereotyping is one type of trait inference based on ES, this finding is consistent with the current findings. Similarly, a previous study demonstrated that the MPFC is engaged in the explicit theory-of-mind process, whereas the DLPFC is engaged in implicit theory-of-mind [61]. This distinction is also consistent with the current results. Further evidence has shown that the MPFC is involved in the explicit aspects of theory of mind. This region was activated more when subjects believed they were playing games with a human opponent compared with a computer program [62], [63]. Similarly, this region is known to be activated more in children than in adults when understanding others' minds, reflecting a greater explicit process in children [64].

Finally, the left PHC is engaged in the explicit ES process. This seemingly contradicts Bar *et al*.'s findings that the PHC is recruited during contextual processing of situation-related objects in the implicit task [21]. However, in the same study, the posterior part of the PHC processed place-related contextual information (e.g., a farm), whereas the anterior part processed more complex contextual information (e.g., a birthday), which is similar to the current findings. This anterior part was activated only when subjects were explicitly instructed to process such contextual information [21]. Actually, this anterior part was comparable to the region identified in this study; hence, the findings of Bar *et al*. are consistent with the present results overall. In short, the implicit/explicit ES network suggested in this study is well supported by previous reports and will enable generation of an overview of this phenomenon.

### The left TPJ and future prediction

The present results suggest that the left TPJ plays an important role in representing hypothetical situations, which characterizes future prediction. Because predicting future situations from fractionally perceived environmental information is essential to cope with a dynamically changing environment, clarifying the neural basis of such a process has great significance. Our suggestion is supported by the fact that the left TPJ was consistently involved in representing hypothetical perspective or perspective differences in previous studies on TOM, perspective taking, and self-transcendence [45]–[51]. Interestingly, in the present study, left TPJ activation was also detected as an explicit ES process-related region, along with other regions such as the MPFC, TP, and PCC. We are not certain if this explicit ES process-related left TPJ is exactly the same region as the left TPJ we found to be future prediction-specific. If the explicit ES-process and future prediction recruit the same left TPJ region, it could be that the left TPJ basically engages in explicit ES, and when future prediction requires an additional load to represent a hypothetical situation, the left TPJ boosts its activity for perspective processing. In the present study, we observed future prediction-specific activity in the left but not right TPJ. This kind of left dominance has been reported in relation to the linguistic nature of the task in previous studies on perspective processing [49], [50]. In the present study, targets of the matching task were presented in words, so it is possible that after the subject inferred a future situation from object pictures they sought the proper word (i.e., name) for that situation. This linguistic load of the task could account for the TPJ's left dominancy in the present study.

Given that subjects were less accurate and had a longer RT in the Ftr condition than in the Exp condition, one might think that the left TPJ activity identified with the contrast (Ftr – Exp) simply reflects the effect of general task difficulty or cognitive load which was relatively greater in the Ftr condition. However, we think this is unlikely because of the following reasons. First, we directly showed that activity in the left TPJ was not correlated with RTs, which reflect general cognitive load common to both the Ftr and Exp conditions, by conducting an additional parametric modulation analysis. Furthermore, previous studies showed that TPJ activity reflecting general cognitive load, such as attention or control demand, is right dominant [65]–[67]. So, if the left TPJ activity in our study reflected an increase in general cognitive load in the Ftr condition, it would be unlikely for us to not to also observe this activity in the right TPJ.

As subjects were required to match the future situation they inferred from object pictures to target in the Ftr condition task, one might think that the left TPJ activity observed in this condition would reflect subjects' intention to infer a future situation that the experimenter had in mind. However, we think that this is unlikely due to the following reasons. First, our task did not require subjects to hit the correct answer in the first place, and did not provide any feedback on hits or misses. Thus, subjects' motivation to infer the correct answer that the experimenter had in mind should have been very low. Second, if some of the subjects were inclined to infer how the experimenter labeled stimulus situations, then this should also be true in the Exp condition. Thus, it is unlikely that such a process is reflected in the activation derived from the comparison (Ftr – Exp).

## Conclusions

In conclusion, these findings show that implicit and explicit ES processes have different neural substrates and that the left TPJ plays an important role in the future-prediction process of ES. It is assumed that the implicit, explicit, and future-prediction sub-processes and their underlying neural substrates collaborate to support ES. Because ES is thought to provide a common basis for various cognitive processes ranging from simple perceptual prediction to complex social cognition, such as theory of mind, these findings enrich the understanding of the neural substrates of the implicit/explicit/predictive aspects of those cognitive processes. Moreover, it is proposed that these results provide a good reference point for future studies that aim to elucidate a unified explanation for these cognitive processes from the viewpoint of neuroscience.

## Supporting Information

Figure S1 **Brain regions specific to explicit event simulation (ES) processing in social events and those in non-social events.** The result is thresholded at p<0.005 without multiple comparisons. (a) Social events: results of the contrast (Exp_social – Imp_social) are shown. (b) Non-social events: results from the contrast (Exp_nonsocial – Imp_nonsocial) are shown. R: right. L: Left. The coordinates are indicated in the Montreal Neurological Institute (MNI) standard space.
(TIF)
