# Supplementary figures and images for: The Neural Basis of Event Simulation: An fMRI Study
Source: PLoS One. 2014 May 2;9(5):e96534. doi: 10.1371/journal.pone.0096534 (PMC4008581; doi:10.1371/journal.pone.0096534)

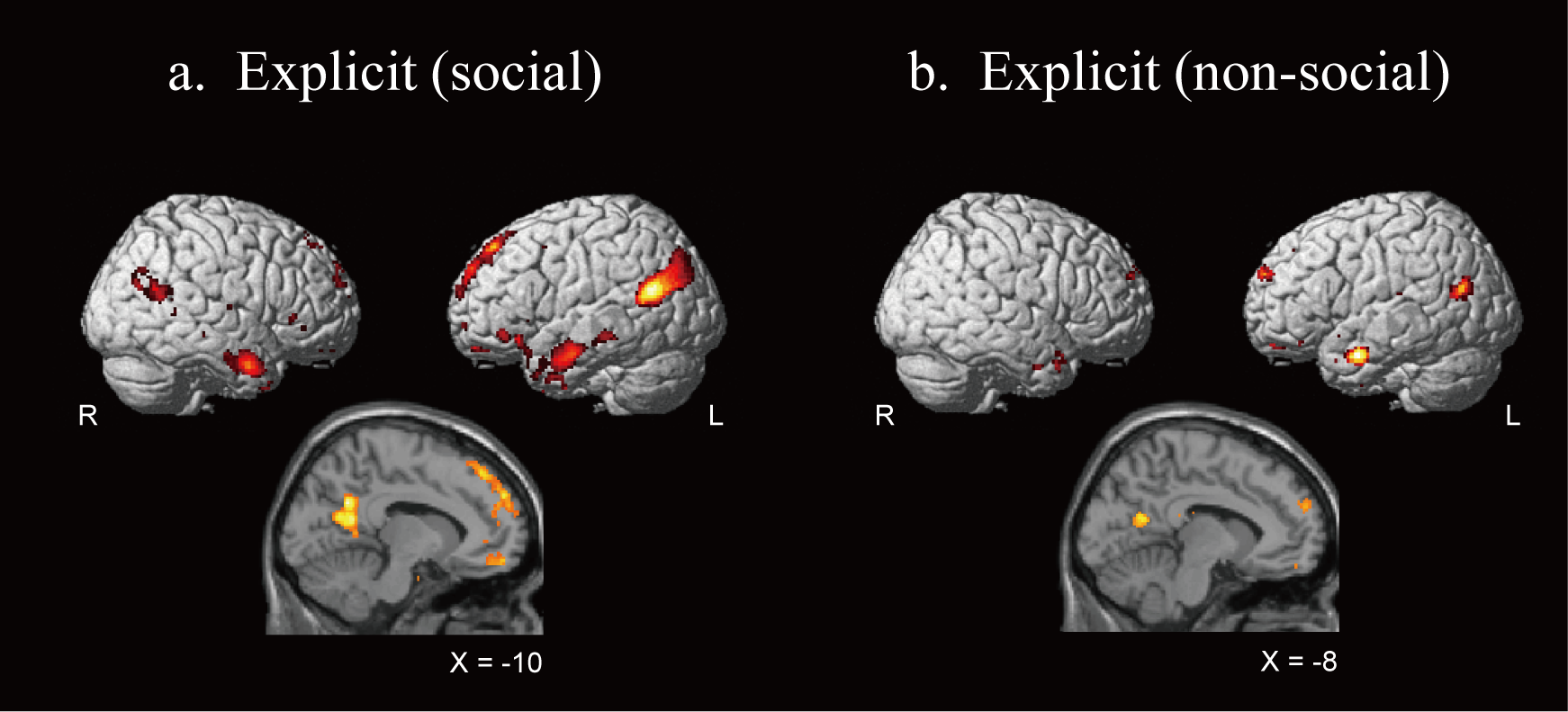

Supplement: Figure S1 — Brain regions specific to explicit event simulation (ES) processing in social events and those in non-social events. The result is thresholded at p<0.005 without multiple comparisons. (a) Social events: results of the contrast (Exp_social – Imp_social) are shown. (b) Non-social events: results from the contrast (Exp_nonsocial – Imp_nonsocial) are shown. R: right. L: Left. The coordinates are indicated in the Montreal Neurological Institute (MNI) standard space. (TIF) [file pone.0096534.s001.tif]
